# SPECT/NIRF Dual Modality Imaging for Detection of Intraperitoneal Colon Tumor with an Avidin/Biotin Pretargeting System

**DOI:** 10.1038/srep18905

**Published:** 2016-01-06

**Authors:** Chengyan Dong, Sujuan Yang, Jiyun Shi, Huiyun Zhao, Lijun Zhong, Zhaofei Liu, Bing Jia, Fan Wang

**Affiliations:** 1 Medical Isotopes Research Center and Department of Radiation Medicine, School of Basic Medical Sciences, Peking University, Beijing 100191, China; 2 Interdisciplinary Laboratory, Institute of Biophysics, Chinese Academy of Sciences, Beijing 100101, China; 3 Medical and Healthy Analytical Center, Peking University, Beijing 100191, China; 4 State Key Laboratory of Natural and Biomimetic Drugs, Center for Molecular and Translational Medicine, Peking University, Beijing 100191, China

## Abstract

We describe herein dual-modality imaging of intraperitoneal colon tumor using an avidin/biotin pretargeting system. A novel dual-modality probe, ^99m^Tc-HYNIC-lys(Cy5.5)-PEG_4_-biotin, was designed, synthesized and characterized. Single-photon emission computed tomography/ computed tomography (SPECT/CT) imaging and near infrared fluorescence (NIRF) imaging were developed using intraperitoneal LS180 human colon adenocarcinoma xenografts. Following avidin preinjection for 4 hours, ^99m^Tc-HYNIC-lys(Cy5.5)-PEG_4_-biotin could successfully detect colon tumors of different sizes inside the abdominal region using both modalities, and the imaging results showed no differences. Biodistribution studies demonstrated that the tumors had a very high uptake of the probe ^99m^Tc-HYNIC-lys(Cy5.5)-PEG_4_-biotin (12.74 ± 1.89% ID/g at 2 h p.i.), and the clearance from blood and other normal tissues occured very fast. The low tumor uptake in the non-pretargeted mice (1.63 ± 0.50% ID/g at 2 h p.i.) and tumor cell staining results showed excellent tumor binding specificity of the pretargeting system. The ability of the novel probe to show excellent imaging quality with high tumor-to-background contrast, a high degree of binding specificity with tumors and excellent *in vivo* biodistribution pharmacokinetics should prove that the avidin/biotin based dual-modality pretargeting probe is a promising imaging tool during the entire period of tumor diagnosis and treatment.

Surgery combined with chemotherapy is regarded as the most effective treatment for cancer patients^1^. To adequately discriminate between tumor and normal tissues and determine the tumor-free margin are the key demands of oncologic surgery for a surgeon^2^. With intraoperative, tumor-specific detection supplied by imaging modalities, surgeons in surgery could localize the tumor lesion specifically, leading to more radical excision of tumor tissue that could effectively protect the function of adjacent normal tissues and offer an improved prognosis. Image-guided surgery using appropriate probes could assist surgeons by providing real-time feedback with visual observation and palpation^3^. Among the imaging modalities investigated to date, optical imaging, particularly near-infrared fluorescence (NIRF; 700- to 900-nm wavelength) imaging, is clearly perfectly suited for image-guided surgery due to its superior resolution and sensitivity, relatively low tissue absorption and scattering, as well as minimal autofluorescence of NIR light in this spectrum^4^. Many preclinical and clinical studies have demonstrated the potential use of NIRF imaging in image-guided surgery. Recently, Gooitzen M reported the first-in-human use of intraoperative tumor-specific fluorescence imaging for real-time surgical visualization of tumor tissue in patients with ovarian cancer^1^.

However, the limited tissue penetration of NIRF imaging leads to low detection in intraperitoneal tumors before surgery. Single-photon emission computed tomography (SPECT) with its high penetration ability and sensitivity has been used routinely for clinical applications in the early detection of lesions, staging malignant tumors, and the evaluation of therapeutic efficacy^5^. In the present study, we developed a simultaneous optical and nuclear contrast agent, the dual-function SPECT/NIRF probe, which could be used for detecting tumor effectively during the entire period of the diagnosis and therapy, even in image-guided surgery.

To improve the application potential of the novel probe, we designed a two-step pretargeting approach using a biotin/avidin interaction system, resulting in enhanced tumor-to-nontumor ratios and modest signal amplification at the tumor site due to the tetrameric architecture of avidin for biotin^6^. Pretargeting supports an optimal targeting process, which first localizes the tumor tissue using an unlabeled bispecific molecule before administering a small fast-clearing signal compound. The combination of high specific uptake in tumors and low retention in normal tissues that the pretargeting system brings could affect the image-guide surgery, which relies on the maximized contrast between the tumor and non-tumor tissues^7^. Moreover, we embed the tetra (ethylene glycol) (PEG_4_) spacer between the biotin motif and signal motif to improve the pharmacokinetics *in vivo*^8^.

## Results

### Synthesis of HYNIC-lysine(Cy5.5)-PEG_4_-biotin

**Figure 1:**
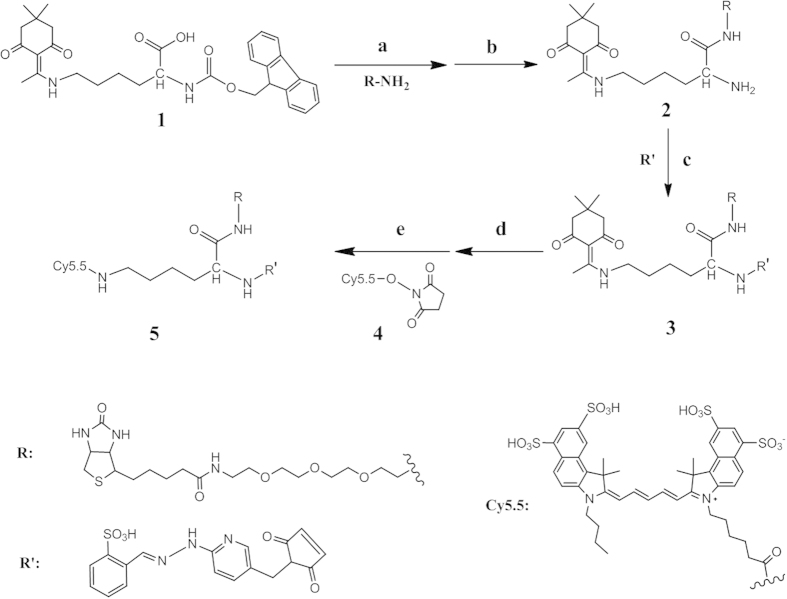
Synthesis of HYNIC-lys(Cy5.5)-PEG_4_-biotin. Reagents and conditions: (**a**) EDC, Sulfo-NHS, DMF:H_2_O(1:1), 28 °C, 10 h, 52.1%; (**b**) 20% piperidine, DMF, rt, 0.5 h, 82.9%; (**c**) H_2_O:DMF =1:1, DIPEA, pH 8.5, 28 °C, 10 h, 58.2%; (**d**) 5% Hydrazine, DMF, rt, 0.5 h, 87.0%; (**e**) 0.1 M Na_2_B_4_O_7_, pH 8.5, dark, r.t, 10 h, 52.1%.

To make the probe bi-functional, protected lysine was applied in this synthesis. HYNIC-lys(Cy5.5)-PEG_4_-biotin was prepared in five steps (Fig. 1), with an overall yield of 11.4%, starting from PEG modified biotin, **R-NH**_**2**_. **R-NH**_**2**_ was coupled with Dde and Fmoc protected lysine (**1**), and subsequently deprotected in 20% piperidine in DMF to yield **2**. The introduction of the chelator HYNIC group used for ^99m^Tc labeling was accomplished by coupling with s-SBZ-HYNIC, followed by deprotection of the Dde groups with 5% hydrazine in DMF. The optical Cy5.5 region (**4**) was linked to the remaining N-terminal region of compound **3**, and the final product compound **5** was prepared. In all of the synthetic steps, the compounds were purified and characterized by reversed-phase preparative HPLC. All of the new conjugates were analyzed by both HPLC and mass spectrometry to confirm the identity of the products (See Methods and Supplementary Figures). The HPLC purity of the final product was >95% before being used for ^99m^Tc-labeling.

### Radiochemistry

^99m^Tc-HYNIC-lys(Cy5.5)-PEG_4_-biotin was prepared using a non-stannous formulation with tricine and TPPTS because TPPTS is a reducing agent for Na[^99m^TcO_4_]. After radiolabeling, the radiochemical purity of the ^99m^Tc-labeled probe was >98% as measured by radio-HPLC. The solution stability of ^99m^Tc-HYNIC-lys(Cy5.5)-PEG_4_-biotin was studied by radio-HPLC in saline and in the presence of excess cysteine (1.0 mg/mL, pH = 7.4), and the radiotracer was stable in the saline and cysteine solution for >6 h.

### Dual-modality imaging

**Figure 2:**
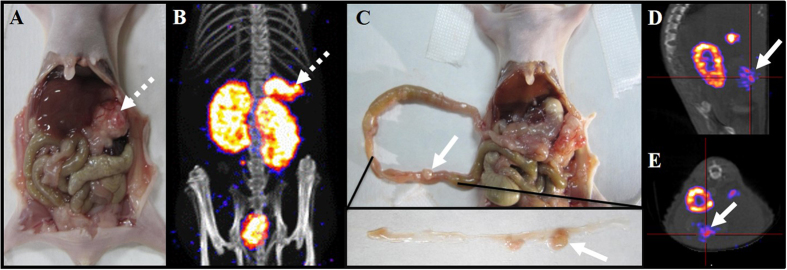
SPECT/CT imaging of ^99m^Tc-HYNIC-lys(Cy5.5)-PEG_4_-biotin in nude mice bearing LS180 colon tumor. (**A**,**B**) Dashed arrows mark location of LS180 tumors of large size among the liver, stomach, spleen and pancreas. (**C**,**D**,**E**) The solid arrows mark the location of LS180 tumors of small size along the intestine wall.

We tested the application of the probe in the LS180 colon tumor model. Nude mice were intraperitoneally injected with 1 × 10^6^ of cultured LS180 cells. It takes 10 days to obtain colon tumors of the appropriate size in the animal model (Fig. 2A,C). Based on the pretargeting strategy, the tumor-bearing animals were pretreated with avidin (15 μg/g whole body weight) for 4 h prior to the radiolabeled probe injection, followed by administration of 100 μL of the labeled conjugate **5** (18.5 MBq)^9^. The imaging of the colon tumor model was carried out after 2 h of radiotracer injection using two imaging modalities. SPECT/CT and image analysis were performed using a rodent scanner (NanoSPECT/CT; Mediso Ltd.). As shown in Fig. 2B, tumors in the stomach, intestine, spleen and pancreas were clearly visualized (white arrow), and the activity level in the abdominal region except the kidneys and bladder (particularly liver and intestine) was very low. In addition, some small-sized tumors could be distinguished very clearly (Fig. 2C–E). The high tumor uptake and superior tumor-to-background ratios make it possible for tumor detection in the diagnosis before surgery and lesion recheck in treatment evaluation after surgery.

**Figure 3:**
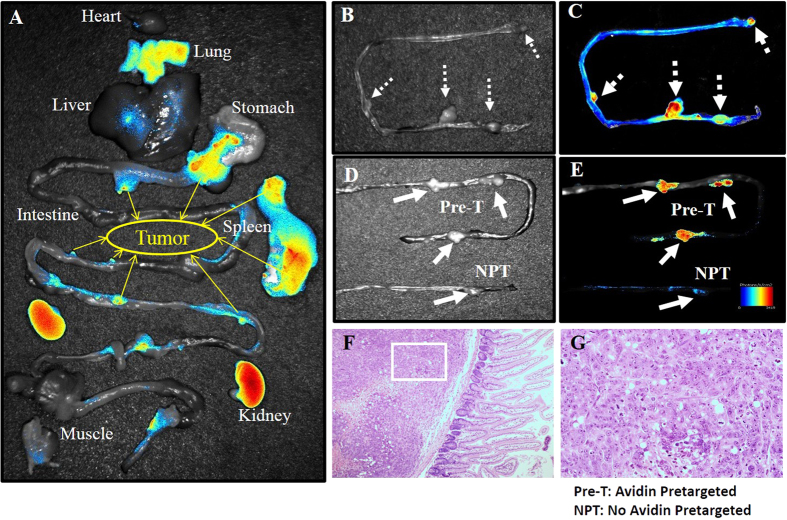
NIR-fluorescence imaging of ^99m^Tc-HYNIC-lys(Cy5.5)-PEG_4_-biotin in nude mice bearing LS180 colon tumors. (**A**) Tissue uptake of HYNIC-lys(Cy5.5)-PEG_4_-biotin. Yellow arrows mark tumors. (**B**,**C**) Local NIRF imaging of mouse intestines with colon tumors. Dashed arrows mark tumors. (**D**,**E**) Contrast between tumor uptakes of HYNIC-lys(Cy5.5)-PEG_4_-biotin with and without pretargeted avidin. Solid line arrows mark the tumors. (**F**,**G**) HE staining of selected tumor section.

Figure 3A displays NIRF imaging of all of the major organs with the dual-modality probe using Maestro2 (Perkin Elmer). The tumors of different sizes could be well observed due to the high tumor uptake, and the uptake of normal tissues in the abdominal region except the kidneys was relatively low. As shown in Fig. 3B,C, four colon tumors were co-localized very well between the photograph and NIRF image. To test the effectiveness of the pretargeting system, LS180 colon tumor-bearing nude mice were injected with the same dose of ^99m^Tc-HYNIC-lys(Cy5.5)-PEG_4_-biotin and vehicle saline instead of avidin pretargeting. The contrast results were shown in Fig. 3D,E. The tumor uptake of the pretargeting group was significantly higher than that of the non-pretargeting group, indicating that endogenic avidin exists at a very low concentration in normal tissues and affects the imaging quality very negligibly. The hematoxylin and eosin (H&E) staining of selected tissue sections identified the high biotin-based probe uptake region consistent with the tumor region. The results are shown in Fig. 3F,G. Figure 3 demonstrates the tumor location and margin status in the colon tumor model. The tumors grown along the intestine wall could be easily detected by NIRF imaging because of the high tumor-to-intestine ratio.

### Biodistribution Characteristics

**Figure 4:**
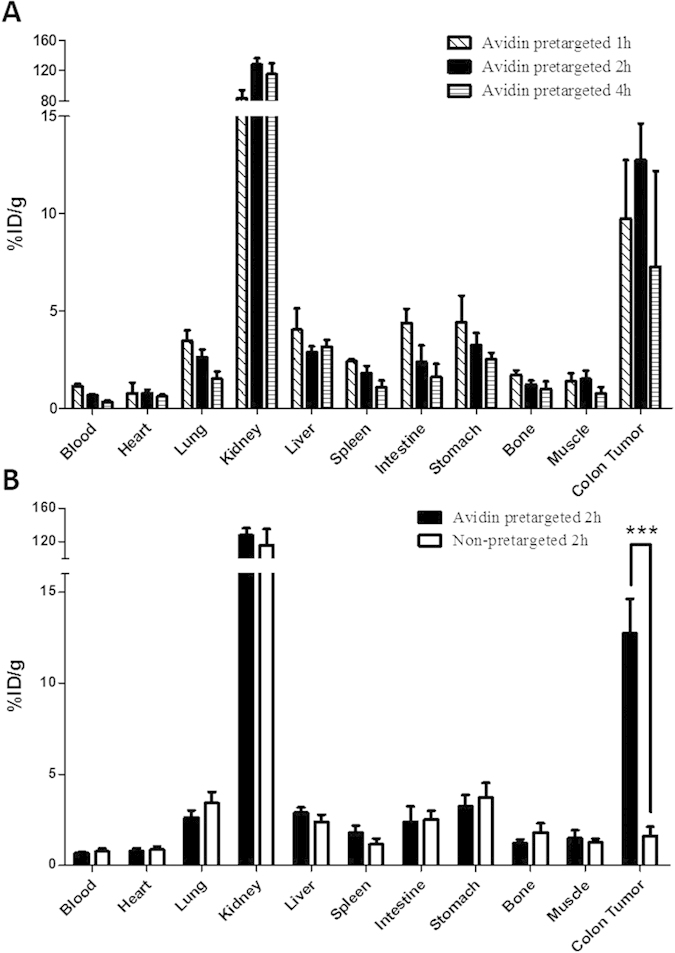
Biodistribution of ^99m^Tc-HYNIC-lys(Cy5.5)-PEG_4_-biotin in nude mice bearing LS180 colon tumors. (**A**) Biodistribution of the probe at 1 h, 2 h and 4 h p.i. (**B**) Biodistribution of the probe with or without pretargeted avidin.

The nude mice bearing LS180 xenografts were used to evaluate the biodistribution characteristics and excretion kinetics of ^99m^Tc-HYNIC-lys(Cy5.5)-PEG_4_-biotin. In general, ^99m^Tc-HYNIC-lys(Cy5.5)-PEG_4_-biotin showed a promising distribution in the tumor-bearing model (Fig. 4). The radiolabeled probe displayed a rapid clearance predominantly via the renal route as evidenced by higher kidney uptake and a rapid blood washout (1.14 ± 0.13%ID/g and 0.32 ± 0.09%ID/g at 1 and 4 h p.i., respectively). The intestines and other dissected tissues except the kidneys showed very low uptake, a finding that was also consistent with the imaging results. The LS180 tumor uptake was as high as 9.76 ± 2.99%ID/g at 1 h p.i., then increased to 12.74 ± 1.89%ID/g at 2 h p.i.

To test the tumor-targeting specificity of avidin, the biodistribution of non-pretargeted mice was performed with the results shown in Fig. 4B. The tumor uptake at 2 h p.i. was 1.63 ± 0.50%ID/g without avidin pretargeting, indicating that the specific targeting of avidin in LS180 tumors and endogenic avidin did not affect the tumor uptake. The uptakes of other normal tissues did not change in both groups, further demonstrating the tumor-targeting specificity of avidin.

### Cell Staining

**Figure 5:**
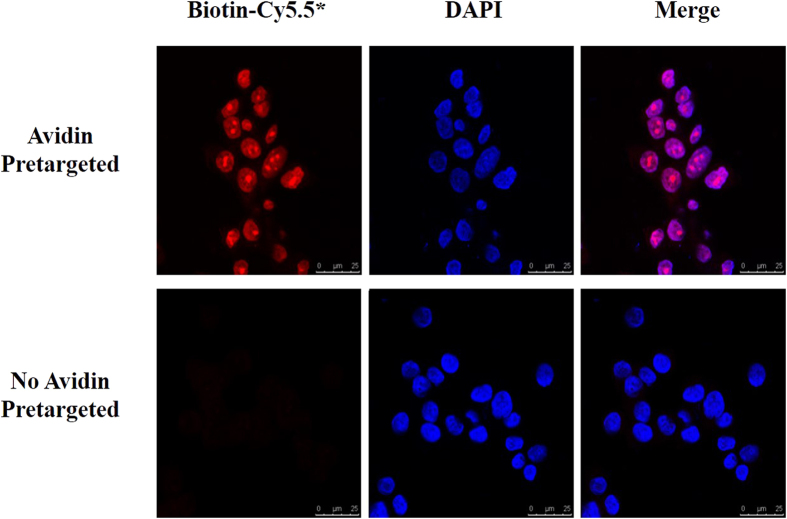
Fluorescent staining of LS180 cells with HYNIC-lys(Cy5.5)-PEG_4_-biotin (red) and DAPI (blue). * means HYNIC-lys(Cy5.5)-PEG_4_-biotin.

We utilized *ex-vivo* microscopy to visualize the location of avidin in LS180 cells. Cells were incubated for 2 hours at 37 °C with avidin followed by incubation with HYNIC-lys(Cy5.5)-PEG_4_-biotin at room temperature (RT) for 1 h. Nuclei were labeled with DAPI for 5 min at RT. Immunofluorescence staining showed that the avidin could bind to the tumor cells and most of the avidin accumulated in the nuclear region (Fig. 5).

## Discussion

We reported the synthesis and characterization of a novel pretargeting dual-labeled imaging probe that contains an NIR dye (Cy5.5) for fluorescence optical imaging and a nuclide (^99m^Tc) for nuclear imaging. Lysine was used to link these two motifs to the avidin-targeting molecule biotin (Fig. 1). Furthermore, we embedded a PEG_4_ spacer between the biotin motif and signal motifs to improve the pharmacokinetics *in vivo* and reduce steric hindrance of biotin/avidin binding. To achieve a better imaging result, we first combined the two concepts of “dual-modality imaging” and “pretargeting”.

The pretargeting concept was proposed by Reardan *et al*.^10^ as an alternative to coupling a chelate directly to antibodies in 1985. This promising concept quickly led to the development of radioimmunotherapy (RIT), overcoming the limitation of the slow blood clearance of directly radiolabeled tumor-targeted antibodies. Pretargeting involves two steps in which the pretargeting agents localize in the tumor first, and then the radiolabeled compounds bind to the pretargeting agents^11^,^12^. The uptake of the normal tissues, particularly the liver, could decline after the clearance. Preclinical studies have already shown that tumor detection of the pretargeting probes with many radionuclides (^18^F, ^124^I, ^64^Cu and ^68^Ga for PET imaging; ^99m^Tc, ^131^I, and ^111^In for SPECT imaging) is effectively more excellent than detection using directly radiolabeled peptide probes or even directly radiolabeled antibodies^13^,^14^,^15^,^16^,^17^,^18^.

In our study, biotin-avidin binding-based method was used for two reasons: their very high affinity (10^−15^M) and superior signal amplification with four binding sites for biotin per avidin. This biotin/avidin pretargeting system has been proven to be a successful approach to tumor imaging and radioimmunotherapy by many preclinical and clinical studies. Avidin is also a tumor-targeted molecule^19^ that has also been reported to bind to glycoprotein-binding receptors (lectins) on cancer cells and facilitate internalization into cancer cells. The presence of endogenous lectins with galactoside-binding specificities has been reported in various tumor cells, and their expression leads to a higher metastatic potential. Moreover, the high glycosylation and high pI of avidin may increase their binding properties^20^. Figure 5 clearly demonstrates that avidin could bind to LS180 cells specifically and localize in the cell nuclei region. Interestingly, we noticed a distinctive morphological feature—morular structures—in the nuclei because nuclear biotin inclusions named biotin-rich optically clear nuclei (BROCN) are demonstrated in certain tumors, including low-grade adenocarcinoma of the fetal lung type (L-FLAC)/well-differentiated fetal adenocarcinoma (WDFA), cribriform-morular variant of papillary thyroid carcinoma (CMV), adenoma of the gallbladder, adenoma of the colon, pancreatoblastoma, endometrioid tumor of the ovary, and adenoacanthoma of the uterus^21^.

The avidin/biotin pretargeting system could successfully detect tumors in both imaging modalities (Figs 2 and 3). The tumor model was established via the intraperitoneal injection of cultured LS180 human colon adenocarcinoma cells. Two types of tumors could be observed in the abdominal region. Larger tumors grew in the stomach, pancreas and spleen (Fig. 2A, dashed white arrow), and smaller tumors were shown along the intestine wall (Fig. 2C, solid white arrows). Figure 2 illustrates the SPECT/CT imaging of the new probe ^99m^Tc-HYNIC-lys(Cy5.5)-PEG_4_-biotin in LS180 tumor-bearing nude mice. The tumors of different sizes could be clearly seen in the images (Fig. 2B for the larger size; Fig. 2D,E for the smaller sizes). The pretargeted ^99m^Tc radiotracer only accumulates in the kidney and tumor lesion, even in the small ones. Combined with the biodistribution shown in Fig. 4, ^99m^Tc-HYNIC-lys(Cy5.5)-PEG_4_-biotin is accumulated in tumors rapidly (9.76 ± 2.99%ID/g at 1 h p.i.) and cleared from the blood and other normal tissues very quickly (1.14 ± 0.13%ID/g in blood, 4.04 ± 1.09%ID/g in liver, and 2.42 ± 0.11%ID/g in spleen at 1 h p.i.). The tumor achieves a maximum uptake at 2 h p.i. (12.74 ± 1.89%ID/g). Tumor/blood ratios of 8.23 ± 2.21 at 1 h p.i. to as high as 18.98 ± 4.38 at 2 h p.i. were obtained to support excellent tumor imaging. A high tumor/tissue ratio within 2 h is very beneficial for clinical imaging purposes because many radionuclides with short half-lives could be used to reduce the radiation damage to the normal tissues of the patients (Supplementary Table 1). ^99m^Tc is an important radionuclide for widespread imaging applications with ideal nuclear properties, including a short half-life (*t*_*1/2*_ = 6 h), rich and diverse coordination chemistry, high specific activity (single photon emission at 140 keV), and easy availability at low cost from a commercial generator. In our study, the radiolabeling process was very easy, and the labeling yield was as high as 98%. Tricine and the reducing agent TPPTS are used as the coligands, and stannous chloride is not needed. It is not complicated to develop a kit formulation for routine preparation of the radiotracer.

There is no single ideal modality for all experimental purposes^22^,^23^. Each modality has its own advantages and disadvantages. Nuclear imaging offers good sensitivity at deep tissue sites and could detect orthotopic tumors and their metastatic lesions^24^. Additionally, optical imaging offers real-time and high-resolution imaging of fluorophores in tumors^25^. For image-guided surgery, NIRF-based imaging is preferred because of the relatively low tissue absorption and minimal autofluorescence of NIR light. In this study, we developed an *ex vivo* optical imaging instead of an *in vivo* imaging. Figure 3A shows the accumulation of the fluorescence in major organs. An optical image was overlaid on a white-light image for easy identification. At 2 h p.i., the fluorescence signal intensities were mainly attributable to the kidneys and tumors. Tumors of different sizes could be clearly distinguished (yellow solid arrows). The optical imaging data are consistent with nuclear imaging data. In clinic, SPECT/CT imaging can supply the situation about the tumor before the surgery, including the size, location and metastasis in the distant organs. With the accurate information, the surgery will be guided with NIRF imaging for a more radical excision.

Local NIRF imaging of mouse intestines with colon tumors showed more promising results (Fig. 3B,C). All four tumors in the field could be imaged very clearly (white dashed arrows). HE staining of selected sections was performed to prove that the fluorescence probe accumulated in the tumor region (Fig. 3F,G). To investigate the specificity of avidin to LS180 tumor, we tested the contrast for the tumor uptake of HYNIC-lys(Cy5.5)-PEG_4_-biotin with and without pretargeted avidin (Fig. 3D,E). Without avidin pretargeting, the tumor uptake was similar to that of the background, significantly lower than the pretargeted tumor uptake. Figure 4B demonstrated that the tumor uptake of the pretargeting group at 2 h p.i. (12.74 ± 1.89%ID/g) was significantly higher than that of the non-pretargeting group (1.63 ± 0.50%ID/g). Additionally, there was no significant change in the probe uptake for the two groups in other organs like the heart, intestines, kidneys, lungs, liver and spleen.

The avidin/biotin pretargeting dual-modality system successfully accomplished the purpose of the probe being able to effectively diagnose the tumor before the operation, guide the surgery and evaluate the treatment effect. Although research on avidin targeted diseases is not sufficient, the exciting results illustrate that this system could translate to other peptides and antibodies. By conjugating other targeting molecules with avidin, many receptors on the tumors and other diseases could be localized with the pretargeting molecules, then the receptors could be imaged with the dual-modality probe ^99m^Tc-HYNIC-lys(Cy5.5)-PEG_4_-biotin, which has many superior advantages such as high binding affinity, high specificity, high tumor/non-tumor ratios and fast clearance from normal tissues. The results could be identical to those with SPECT imaging and NIRF imaging, supporting the accuracy of the detection. We will test this system on other receptors to investigate the effectiveness. Based on the expected favorable pharmacokinetic properties, we anticipate that the probe could be applied clinically. However, the doses and imaging time should be determined before translation into the clinic. Moreover, the radiation exposure to surgeons and hospital staff may limit the use of the probe in clinic. Although many studies have showed the radiation exposure to the operating team is only a minor problem for ^99m^Tc-labelled tracers under formal SPECT imaging guided operating procedures^26^,^27^, the accurate absorbed staff doses at operations should be measured where patients had received a preoperative injection of ^99m^Tc-HYNIC-lys(Cy5.5)-PEG_4_-biotin to guarantee the safety of the surgical staff.

In summary, we described the design, synthesis, and characterization of ^99m^Tc-HYNIC-lys(Cy5.5)-PEG_4_-biotin, a novel dual-modality probe containing a HYNIC group for labeling the isotope ^99m^Tc for SPECT imaging, and a Cy5.5 group for NIR fluorescence imaging. This is the first study on the combination of pretargeting and dual-modality imaging. Both modalities successfully detect the colon tumors of different sizes inside the abdominal region. High tumor uptake, high binding affinity, high specificity and low organ accumulation make the probe a promising imaging tool. Employing both nuclear and optical imaging with the pretargeting system may facilitate translation into clinical applications.

## Methods

### Materials and Instruments

Chemicals purchased from commercial sources were of analytical grade or better and were used without further purification. Biotin-PEG_4_-amine (biotin) was obtained from BioMatrik (Jiaxing, China). Fmoc-Ne-1-(4,4-dimethyl-2,6-dioxocyclohex-1-ylidene)ethyl-L-lysine (lysine(-Dde)-Fmoc) was obtained from Ala biochem company (Jiangsu, China). 1-[3-(Dimethylamino)propyl]-3-ethylcarbodiimide hydrochloride (EDC) and trifluoroacetic (TFA) were obtained from Fluka (Switzerland). N-Hydroxyl-succinimide (NHS), N,N-dimethylformamide (DMF), and diethylamine were obtained from Sigma (USA). Acetonitrile was obtained from Bardik & Jackson. Sodium succinimidyl 6-(2-(2-sulfonatobenzaldehyde)hydrazono)nicotinate (HYNIC-NHS) was a gift from Shuang Liu (Purdue University). Na^99m^TcO_4_ was obtained from a commercial ^99^Mo/^99m^Tc generator (Beijing Atom High Tech Co., Ltd., China).

All experimental protocol were approved by Peking University Health Science Center Academic Committee, including radiolabeling, *in vivo* imaging, and biodistribution studies. All methods were carried out in accordance with the approved guidelines.

### HPLC methods

All synthetic reactions were monitored by high-performance liquid chromatography (HPLC). The reversed-phase HPLC system was the same as that previously reported. Reversed-phase HPLC chromatography was performed on an YMC C-18 column (5 μm, 250 × 4.5 mm) using a gradient solvent system at a flow rate of 1 mL/min. A gradient of acetonitrile/0.05% trifluoroacetic (TFA) (phase A) and H_2_O/0.05% TFA (phase B) was used.

HPLC method 1: Starting with 20% acetonitrile/0.05% TFA (phase A), the initial solvent mixture was held for 8 min, followed by a gradient mobile phase from 20% A at 8 min to 80% A at 35 min and to 100% at 40 min. HPLC method 2: Starting with 5% acetonitrile/0.05% TFA (phase A), the initial solvent mixture was held for 8 min, followed by a gradient mobile phase from 5% A at 8 min to 80% A at 35 min and to 100% at 40 min. HPLC method 3: The mobile phase starting from 0% A at 0 min to 80% A at 35 min and to 100% at 40 min. HPLC method 4: Starting with 5% acetonitrile/0.05% TFA (phase A), the initial solvent mixture was held for 8 min, followed by a gradient mobile phase from 5% A at 8 min to 47.2% A at 25 min and to 100% at 30 min. HPLC method 5: Starting with 0% acetonitrile/0.05% TFA (phase A), followed by a gradient mobile phase from 0% A at 0 min to 5% A at 5 min and to 66% at 30 min.

### Synthesis of Fmoc-lys(Dde)-PEG_4_-biotin

Lysine(Dde)-Fmoc (12.92 mg; 24.2 μmol dissolved in 50 μL of DMF), EDC (15.64 mg dissolved in 50 μL of H_2_O), and NHS (9.52 mg dissolved in 50 μL of DMF) were added to PEG_4_-biotin (6.8 mg; 16.2 μmol dissolved in 50 μL of DMF) at room temperature (RT). After stirring for 12 h at RT, the product was purified by HPLC (method 1). Fractions at 28 min were collected. Fmoc-lys(Dde)-PEG_4_-biotin was obtained with a 52% yield and >95% HPLC purity. The matrix-assisted laser desorption/ionization time-of-light mass spectrometry data were as follows: m/z = 933.50 for [M+H]^+^, 955.52 for [M+Na]^+^, and 971.56 for [M+K]^+^ (C_49_H_68_N_6_O_10_S; calculated molecular weight, 932.47).

### Synthesis of NH_2_-lys(Dde)-PEG_4_-biotin

Fmoc-lys(Dde)-PEG_4_-biotin (7.87 mg, 8.4 μmol) was taken in 20% piperidine in DMF (vol:vol). The solution was stirred at RT for 0.5 h, and the product was isolated by HPLC (method 4). Fractions at 17 min were collected. NH_2_-lys(Dde)-PEG_4_-biotin was obtained at 83% yield with >95% HPLC purity. The high-resolution mass spectrometry (HRMS) data were as follows: HRMS(ESI): m/z = 711.41, calculated for C_34_H_58_N_6_O_8_S (M+H)^+^: 711.41.

### Synthesis of HYNIC-lys(Dde)-PEG_4_-biotin

HYNIC-lys(Dde)-PEG_4_-biotin conjugate was prepared as previously described. Briefly, a solution of 7.0 μmol of NH_2_-lys(Dde)-PEG_4_-biotin was mixed with 11.1 μmol of HYNIC-NHS in 1 mL of DMF. The pH was adjusted to 8.5–9.0 using DIEA. After stirring overnight at RT, HYNIC-lys(Dde)-PEG_4_-biotin was isolated by semipreparative HPLC (method 3). Fractions at 23.0 min were collected. HYNIC-lys(Dde)-PEG_4_-biotin was obtained at 57% yield with >95% HPLC purity. The matrix-assisted laser desorption/ionization (MALDI) time-of-light (TOF) mass spectrometry (MS) data were as follows: m/z = 1014.56 for [M+H]^+^, 1036.64 for [M+Na]^+^, and 1052.53 for [M+K]^+^ (C_47_H_67_N_9_O_12_S_2_; calculated molecular weight, 1013.44).

### Synthesis of HYNIC-lys(NH_2_)-PEG_4_-biotin

HYNIC-lys(Dde)-PEG_4_-biotin (4.13 mg, 4.1 μmol) was taken in 5% hydrazine in DMF (vol:vol). The solution was stirred at RT for 0.5 h, and the product was isolated by HPLC (method 4). Fractions at 19.0 min were collected. HYNIC-lys(NH_2_)-PEG_4_-biotin was obtained at 87% yield with >95% HPLC purity. The matrix-assisted laser desorption/ionization time-of-light mass spectrometry data were as follows: m/z = 850.3 for [M+H]^+^, 872.3 for [M+Na]^+^, 888.3 for [M+K]^+^ (C_37_H_55_N_9_O_10_S_2_, calculated molecular weight 849.35).

### Synthesis of HYNIC-lys(Cy5.5)-biotin

A solution of 1.0 mg (1.17 μmol) of HYNIC-lys(NH_2_)-PEG_4_-biotin was mixed with 1.2 mg (1.58 μmol) of NHS-Cy5.5 in 50 μL of 0.1 M Na_2_B_4_O_7_ buffer (pH = 8.5). After stirring overnight in the dark at RT, HYNIC-lys(Cy5.5)-PEG_4_-biotin was isolated by semipreparative HPLC (method 4). Fractions at 18.6 min were collected. HYNIC-lys(Cy5.5)-PEG_4_-biotin was obtained at 52.1% yield with >95% HPLC purity. Matrix-assisted laser desorption/ionization(MALDI) time-of-light (TOF) mass spectrometry (MS) data were as follows: m/z = 1842.3 for [M+H]^+^ (C_47_H_67_N_9_O_12_S_2_, calculated molecular weight 1013.44).

### ^99m^Tc Radiolabeling and Dose Preparation

To a clean vial were added 20 μg of HYNIC-lys(Cy5.5)- PEG4-biotin, 3 mg of TPPTS and 10 mg of tricine in 100 μL of 25 mM succinate buffer (pH 5.0). To the solution was added 100 μL of Na^99m^TcO_4_ solution (~370 MBq). The mixture was heated at 100 °C for 30 min. After heating, the vial was cooled down to room temperature for ~10 min. A sample of the resulting solution was analyzed by radio-HPLC (Method 2) and ITLC^28^,^29^.

Doses for animal studies were prepared by dissolving the purified radiotracers in saline to yield a concentration of 1.85 MBq/mL for the biodistribution study and 185.0 MBq/mL for the planar imaging study. The resulting solutions were filtered with a 0.20-μm Millex-LG filter unit before being injected into animals. Each tumor-bearing mouse was injected with 0.1 mL of the dose solution.

### Cell Culture and Animal Model

LS180 human colon adenocarcinoma cells were obtained from American Type Culture Collection (Manassas, VA) and were cultured in MEM medium with 10% fetal bovine serum (FBS) at 37 °C in a humidified atmosphere containing 5% CO_2_. Female BALB/c nude mice (4–5 weeks of age) were purchased from the Department of Experimental Animal, Peking University Health Science Center. Nude mice were intraperitoneally injected with 1×10^6^ cultured LS180 cells. All of the animal experiments were approved by Peking University Health Science Center Animal Care Committee (Institutional Animal Care and Use Committee at Peking University) and carried out in accordance with the approved guidelines.

### Biodistribution Study

For biodistribution studies, LS180 tumor-bearing mice (20–25 g) were randomly divided into groups of four mice each. Mice (n=12) received a single intraperitoneal (i.p.) administration of 200 μg/mouse of avidin (in 100 μL of saline), and the other four mice were administered with 100 μL of saline. After four hours, each animal was injected intravenously with 185 kBq of ^99m^Tc-HYNIC-lys(Cy5.5)-PEG_4_-biotin. Animals were anesthetized with an intraperitoneal injection of sodium pentobarbital at a dose of 45.0 mg/kg. Mice were sacrificed by cervical dislocation at 1, 2, and 4 h postinjection (p.i.). Blood, heart, liver, spleen, lung, kidney, stomach, intestine, bone, muscle, and tumor were harvested, weighed, and measured for radioactivity in a γ-counter (Wallac 1470–002; Perkin-Elmer, Finland). Organ uptake was calculated as the percentage of the injected dose per gram of tissue (%ID/g). The biodistribution data were reported as an average plus the standard deviation (mean ± SD) based on the results from four animals at each time point.

### Dual-modality Imaging

The γ-imaging study was performed on female BALB/c nude mice bearing the LS180 human colon adenocarcinoma xenografts. Each tumor-bearing mouse was anesthetized with isoflurane and administered an intravenous dose of 200 μg of avidin in 100 μL of saline or saline only. After four hours, the mice were administered an intravenous dose of 18.5 MBq (1 mCi/1 μg) of ^99m^Tc-HYNIC-lys(Cy5.5)-PEG_4_-biotin in 100 μL of saline. Tumor-bearing mice were imaged by nanoSPECT/CT (Mediso Ltd. Hungary). Next, the animals were sacrificed, and optical imaging of major organs was performed using Maestro2 (Perkin Elmer).

### Cell Staining

The LS180 cell staining study was performed with some modifications. Briefly, 70% to 85% confluent tumor cells grown in 35-mm MatTek glass-bottomed culture dishes were fixed using 4% paraformaldehyde for 10 min. After blocking with 10% FBS in PBS for 30 min, the cells were incubated with avidin (5 μg/mL) for 2 h at 37 °C. The cells were then washed with PBS and stained with HYNIC-lys(Cy5.5)-PEG_4_-biotin (2 μg/mL) for 60 min. After the final wash with PBS, the cells were visualized using a Leica TCS-NT confocal microscope (Wetzler).

### Data and Statistical Analysis

The biodistribution data are reported as averages ± standard deviation based on results from four tumor-bearing mice at each time point. Comparisons between the two biodistribution groups were performed using the t-test (GraphPad Prism 5.0, San Diego, CA). A P value less than 0.05 was considered to be statistically significant.

## Additional Information

**How to cite this article**: Dong, C. *et al*. SPECT/NIRF Dual Modality Imaging for Detection of Intraperitoneal Colon Tumor with an Avidin/Biotin Pretargeting System. *Sci. Rep*. **6**, 18905; doi: 10.1038/srep18905 (2016).

## Supplementary Material

Supplementary Information
