# Supplementary material for: SPECT/NIRF Dual Modality Imaging for Detection of Intraperitoneal Colon Tumor with an Avidin/Biotin Pretargeting System
Source: Sci Rep. 2016 Jan 6;6:18905. doi: 10.1038/srep18905 (PMC4702112; doi:10.1038/srep18905)
Supplement: Supplementary Information [file srep18905-s1.pdf]

# SPECT/NIRF Dual Modality Imaging for Detection of Intraperitoneal Colon Tumor with an Avidin/Biotin Pretargeting System

**Chengyan Dong[1,2,+], Sujuan Yang[1,+], Jiyun Shi[2], Huiyun Zhao[1,3], Lijun Zhong[3], Zhaofei Liu[1], Bing Jia[1]*, and Fan Wang[1,2,4]**

[1] Medical Isotopes Research Center and Department of Radiation Medicine, School of Basic Medical Sciences, Peking University, Beijing 100191, China

[2] Interdisciplinary Laboratory, Institute of Biophysics, Chinese Academy of Sciences, Beijing 100101, China

[3] Medical and Healthy Analytical Center, Peking University, Beijing 100191, China

[4] State Key Laboratory of Natural and Biomimetic Drugs, Center for Molecular and Translational Medicine, Peking University, Beijing 100191, China

+ These authors contributed equally to this work

* Corresponding author: Dr. Bing Jia, Medical Isotopes Research Center, Peking University, 38# Xueyuan Road, Beijing 100191, P. R. China. Phone: +86-10-82802871; Fax: +86-10-82801145; Email: jiabing@bjmu.edu.cn.

# Supplementary information

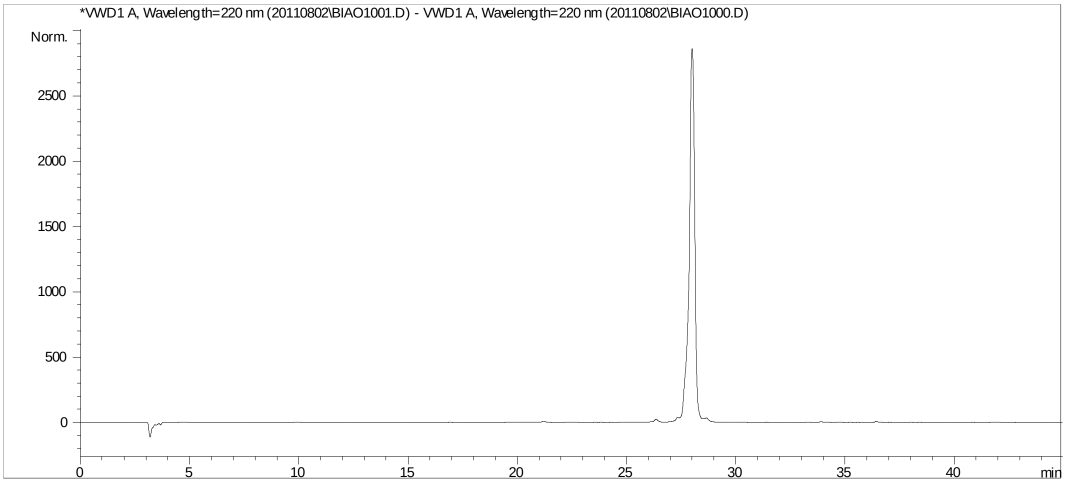


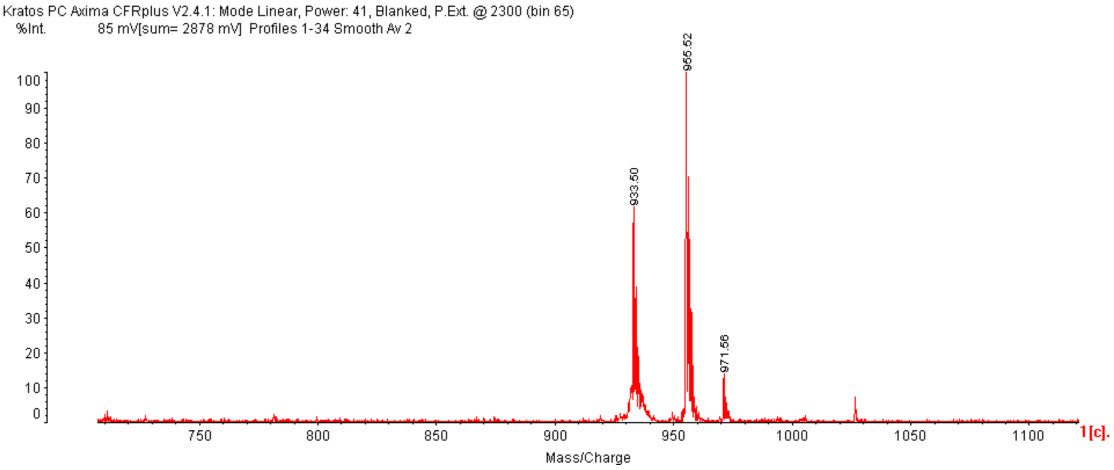


**SFigure.1 HPLC and MS analysis of Biotin-PEG4-lysine(Dde)-Fmoc. HPLC analysis (C18 column): *tR* = 28.2 min, purity > 95%. MALDI-TOF-MS: $[M+H]^+$ m/z 933.5 (calc 933.5); $[M+Na]^+$ m/z 955.5 (calc 955.5); $[M+K]^+$ m/z 971.6 (calc 971.5); MW : 932.5 Yield: 52.1 %.**

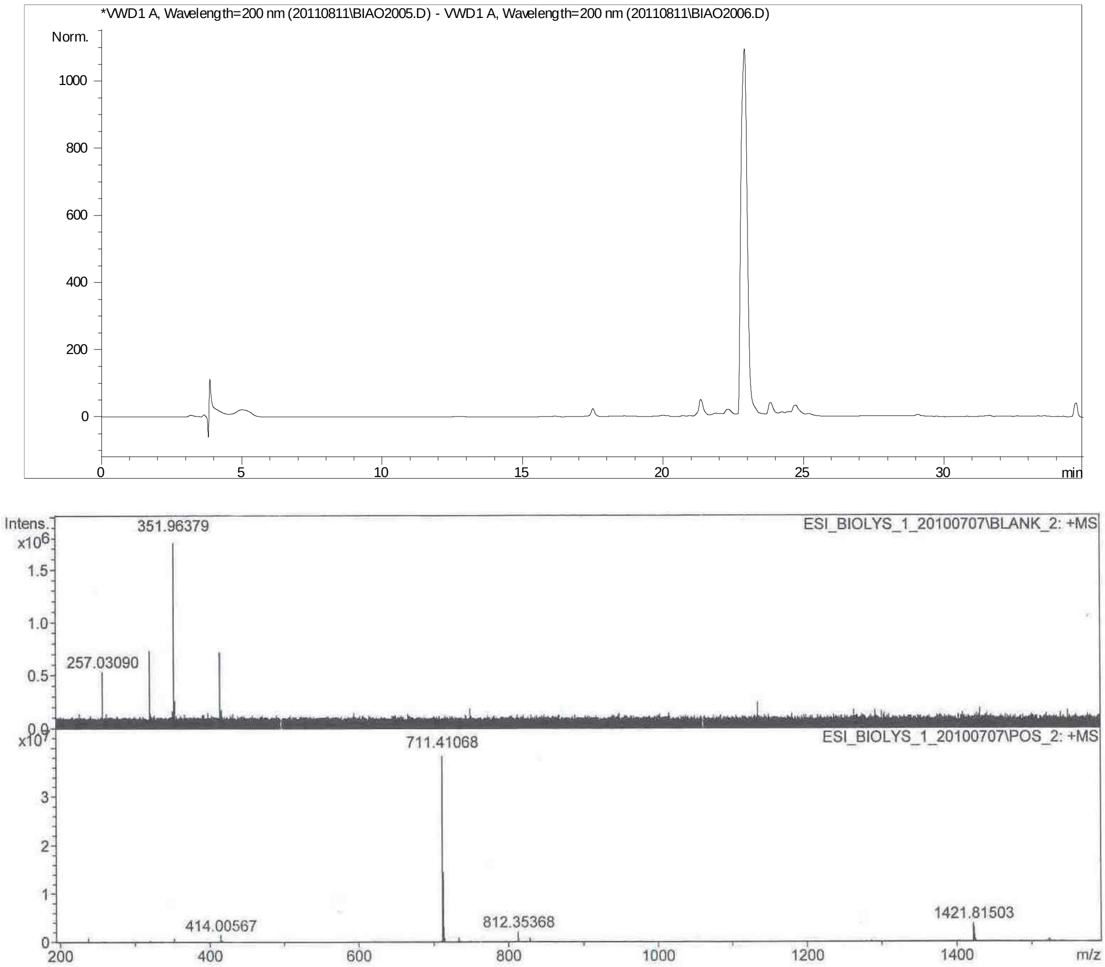


**SFigure.2 HPLC and MS analysis of Biotin-PEG4-lysine(Dde)-$NH_2$. HPLC analysis (C18 column): *tR* = 23.0 min, purity > 95%. ESI-MS: $[M+H]^+$ m/z 711.4 (calc 711.4); MW : 711.4; Yield: 82.9 %.**

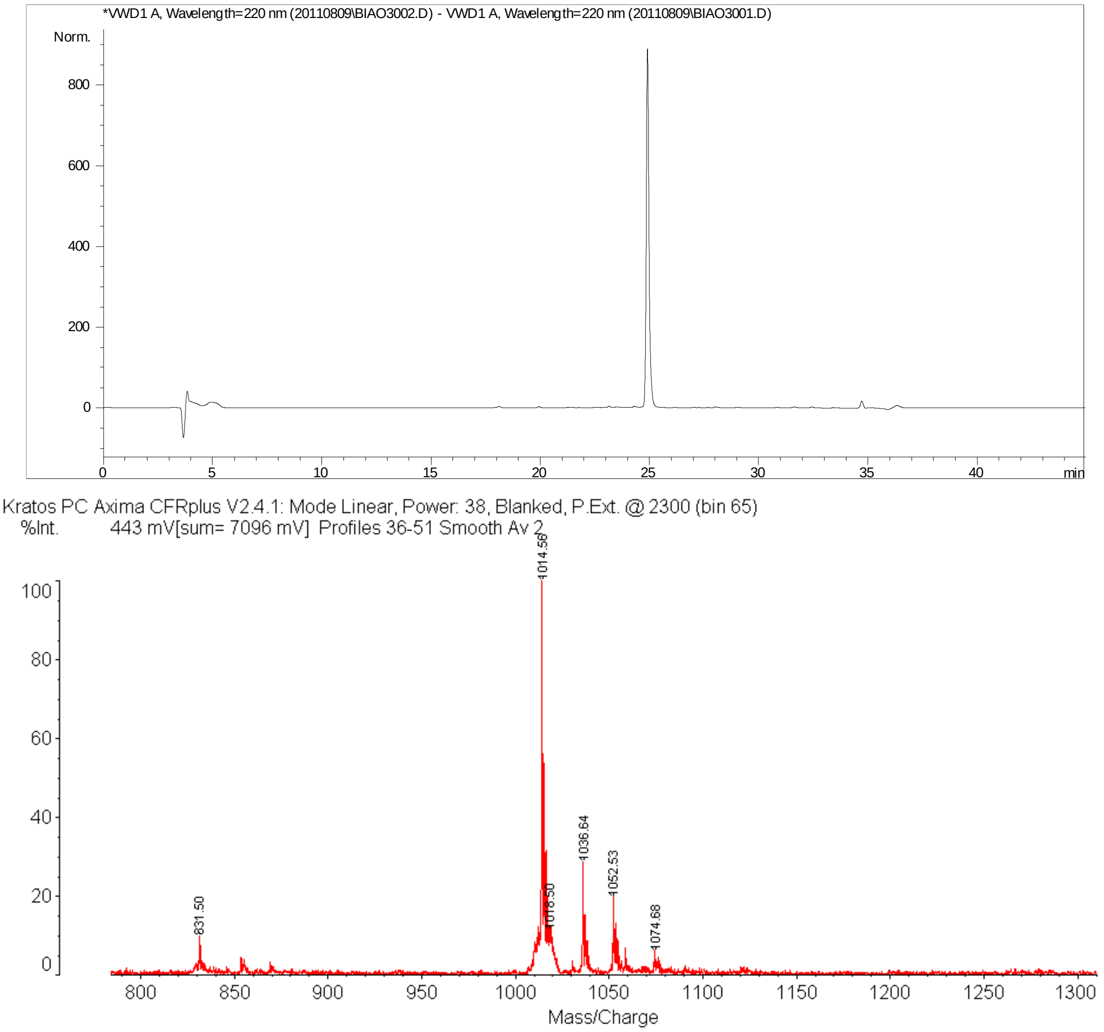


**SFigure.3 HPLC and MS analysis of Biotin-PEG4-lysine(Dde)-HYNIC. HPLC analysis (C18 column): *tR* = 24.9 min, purity > 95%. MALDI-TOF-MS: $[M+H]^+$ m/z 1014.6 (calc 1014.4); $[M+Na]^+$ m/z 1036.6 (calc 1036.4); $[M+K]^+$ m/z 1052.5 (calc 1052.4); MW : 1013.4 Yield: 58.2 %.**

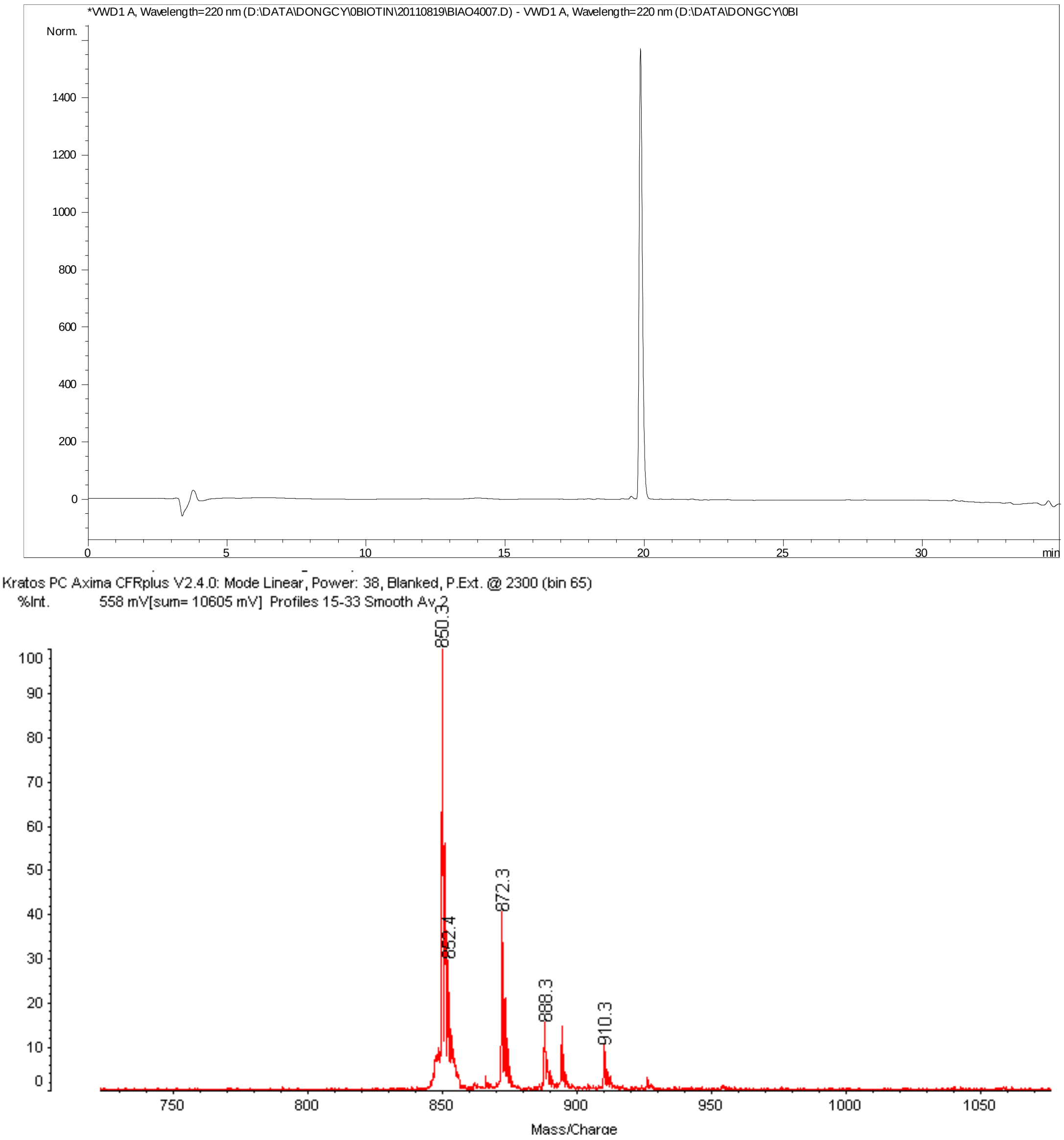


**SFigure.4 HPLC and MS analysis of Biotin-PEG4-lysine($NH_2$)-HYNIC. HPLC analysis (C18 column): *tR* = 20.1 min, purity > 95%. MALDI-TOF-MS: $[M+H]^+$ m/z 850.3 (calc 850.3); $[M+Na]^+$ m/z 872.3 (calc 872.3); $[M+K]^+$ m/z 888.3 (calc 888.3); MW : 849.3; Yield: 87.0 %.**

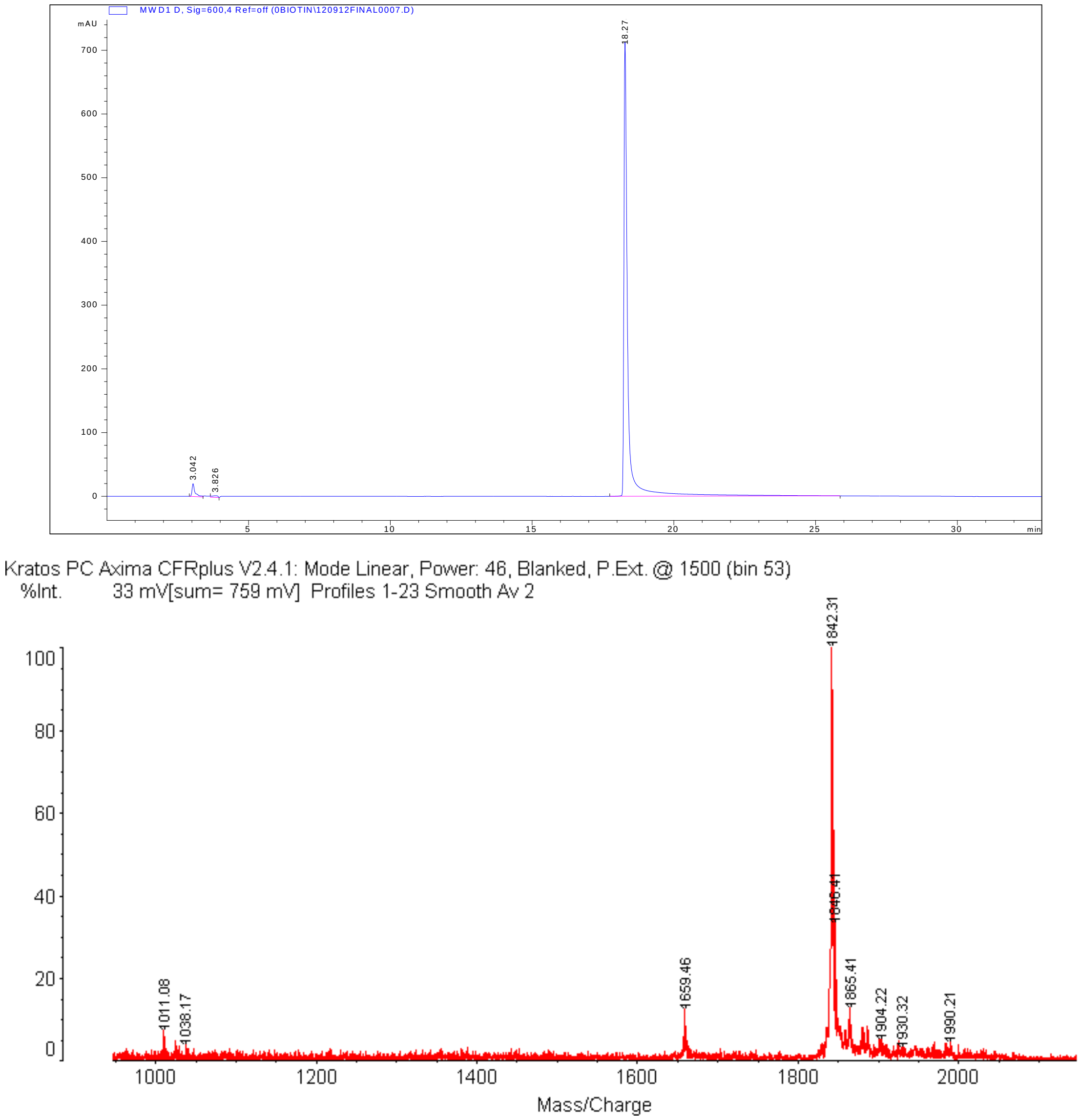


**SFigure.5 HPLC and MS analysis of Biotin-PEG4-lysine(Cy5.5)-HYNIC. HPLC analysis (C18 column): *tR* = 18.6 min, purity > 95%. MALDI-TOF-MS: $[M+H]^+$ m/z 1842.3 (calc 1838.0); MW : 1838.0; Yield: 52.1 %.**

| | 1 h | 2 h | 4 h |
|---|---|---|---|
| Tumor/Blood | 8.58 ± 1.93 | 19.28 ± 3.63 | 21.46 ± 5.78 |
| Tumor/Heart | 9.35 ± 1.06 | 16.62 ± 2.35 | 11.43 ± 4.59 |
| Tumor/Lung | 2.79 ± 0.39 | 4.88 ± 0.41 | 4.60 ± 1.93 |
| Tumor/Kidney | 0.12 ± 0.03 | 0.10 ± 0.02 | 0.07 ± 0.04 |
| Tumor/Liver | 2.62 ± 1.11 | 4.49 ± 1.02 | 2.37 ± 1.38 |
| TumorSpleen | 4.06 ± 1.11 | 7.26 ± 1.53 | 6.86 ± 2.75 |
| Tumor/Intestine | 2.22 ± 0.42 | 5.71 ± 1.62 | 4.46 ± 0.97 |
| Tumor/Stomach | 2.47 ± 1.40 | 3.97 ± 0.51 | 2.78 ± 1.17 |
| Tumor/Bone | 5.71 ± 1.45 | 10.46 ± 0.61 | 7.35 ± 2.05 |
| Tumor/Muscle | 7.20 ± 1.57 | 9.05 ± 3.18 | 9.20 ± 2.48 |

**STable.1 Tumor-to–normal organ ratios of $^{99m}$Tc-HYNIC-lys(Cy5.5)-PEG$_4$-biotin at 1, 2, and 4 hours after pretargeted injection of avidin into nude mice bearing LS180 colon tumors.**
